# Upregulation of heme oxygenase-1 in colorectal cancer patients with increased circulation carbon monoxide levels, potentially affects chemotherapeutic sensitivity

**DOI:** 10.1186/1471-2407-14-436

**Published:** 2014-06-14

**Authors:** Hongzhuan Yin, Jun Fang, Long Liao, Hiroshi Maeda, Qi Su

**Affiliations:** 1 Department of General Surgery, Sheng Jing Hospital, China Medical University, Shenyang City, Liaoning Province 110004, P. R. China; 2 DDS Research Institute, Sojo University, Ikeda 4-22-1, Nishi-Ku, Kumamoto 860-0082, Japan; 3 Laboratory of Microbiology & Oncology, Faculty of Pharmaceutical Sciences, Sojo University, Kumamoto, Japan; 4 School of Public Health, Anhui Medical University, 81th Meishan Road, Hefei City, Anhui Province 230032, P. R. China; 5 Department of Applied Microbial Technology, Faculty of Biotechnology & Life Science, Sojo University, Kumamoto, Japan

**Keywords:** Heme oxygenase-1, Carbon monoxide, Colorectal cancer, Carboxyhemoglobin, Chemosensitivity

## Abstract

**Background:**

Heme oxygenase-1 (HO-1) and its major product carbon monoxide (CO) are known to be involved in the development and progression of many tumors. The present study was to elucidate the expression and function of HO-1 in colorectal cancer (CRC), specially focusing on the circulation CO levels in CRC patients and the possible roles of HO-1 in chemoresistance of colon cancer cells.

**Methods:**

One hundred and eighteen patients received resection for colorectal cancer and polyps at China Medical University Sheng Jing Hospital, were collected in this study. HO-1 expression in CRC tissues was analyzed by immnuohistochemical staining; circulation CO levels as carboxyhemoglobin (COHb) in CRC patients were analyzed by an ABL800 FLEX blood gas analyzer. HO-1 expression in murine colon cells C26 and human colon cancer cells HT29 and DLD1 under HO-1 inducer hemin and anticancer drug pirarubicin (THP) treatment was examined by RT-PCR, and the cell viability after each treatment was investigated by MTT assay. Data were analyzed by student’s t-test or one-way ANOVA followed by Bonferroni t-test or Fisher's exact test.

**Results:**

HO-1 expression in tumor tissues of CRC (61.0%) was significantly higher than in normal colorectal tissues and polyps tissues (29.7%, *P* < 0.01); well-differentiated CRC seemed to express more HO-1 (81.5%) than moderately/poorly-differentiated cancers (59.5%, *P* < 0.05). However, the nuclear HO-1 expression is apparently higher in moderately/poorly differentiated CRC than well-differentiated CRC probably suggesting a new mechanism of function involved in HO-1 in cancer. In parallel with HO-1 expression, circulation CO levels in CRC patients also significantly accelerated. Moreover, HO-1 expression/induction also related to the chemosensitivity of colon cells; HO inhibitor zinc protoporphyrin significantly increased cytotoxicities of THP (i.e., 2.6 – 5.3 folds compared to cells without zinc protoporphyrin treatment).

**Conclusions:**

These findings strongly suggested HO-1/COHb is a useful diagnostic and prognostic indicator for CRC, and inhibition of HO-1 may be a option to enhance the chemotherapeutic effects of conventional anticancer drugs toward CRC.

## Background

Colorectal cancer (CRC) is the third leading cause of cancer-associated death in developed countries [1]. Surgery and combination chemotherapies have been shown to confer only modest survival benefits in advanced CRC, resulting in a five-year survival rate below 10% in patients with metastases to distant organs [2-5]. Recent advances in molecular biology have discovered a wide range of alterations in gene expression during the process of colorectal carcinogenesis. However, the molecular mechanisms by which cancerous development, progression and resistance to chemotherapies occur remain largely unknown.

Heme oxygenase-1 (HO), the antioxidative, antiapoptotic molecule, has attracted great attention in many diseases and disorders including cancer [6-10]. HO is the key enzyme involved in the initial and rate-limiting step in heme degradation, in which heme is degraded and converted to biliverdin, carbon monoxide (CO) and iron is released. Three isoforms of heme oxygenases exist, i.e., HO-1, HO-2 and HO-3, which HO-1 is the inducible form, and recently it is also termed heat-shock protein 32 (Hsp32) [11], whose expression is triggered by diverse stress inducing stimuli including hypoxia, heavy metals, UV radiation, reactive oxygen species (ROS) such as hydrogen peroxide (H_2_O_2_) , and reactive nitrogen oxides such as nitric oxide (NO) [12]. The biological functions of HO-1 are thus believed to be associated with a fundamental adaptive and defensive response against oxidative stress and cellular stress. Ample studies have demonstrated the benefit of HO-1 in a variety of pathological states including vascular injury, hypoxic lung disease and cardiac diseases [13-15]. Regarding cancer, we previously found high HO-1 expression in experimental solid tumors, i.e., rat hepatoma AH136B [16,17] and mouse sarcoma S180 [18]. Administration of HO inhibitor zinc protoporphyrin IX (ZnPP) significantly suppressed the growth of experimental murine solid tumors, which suggests a vital role of HO-1 in tumor growth [16-18]. High expression of HO-1 was also reported in many human tumors in clinic, including brain cancer [19], prostate cancer [11], renal cell carcinoma [20], oral squamous cell carcinoma [21] as well as leukemia [22]. In this context, recently more and more evidence has implicated that HO-1 potentially functions as an important factor associated with the growth and metastasis of tumors, as well as carcinogenesis [10,23,24]. However, the roles of HO-1 in CRC remain to be elucidated.

HO mediated heme degradation is the major source of endogenous CO, which is recently known as a important endogenous gaseous signaling molecule with various biological activities like NO, such as regulation of cell functions by activating soluble guanylate cyclase, involving smooth muscle relaxation and inhibition of platelet aggregation [25-28]. Most functions of HO-1 are now known to be mediated by CO [28]. Suematsu et al. has shown that CO can function as an endogenous modulator of sinusoidal tone in the liver that is an organ with abundant HO-1 [29]. Takeshi Ishikawa et al. have demonstrated that CO can influence cancer metastasis, especially to the liver [30]. Our previous work also indicated that CO might function some effects of HO-1 in tumor growth because addition of bilirubin, another important product of HO-1 catalyzed heme degradation, could not fully reverse the ZnPP-induced apoptosis of AH136B hepatoma cells [17]. We thus hypothesized that endogenous CO may positively reflect the HO-1 level especially in disease conditions such as cancer, serving as the major effector of HO-1 and becoming an indicator of HO-1 expression and functions.

Taking into account these context, we investigated the expression and functions of HO-1 in CRC in clinical manifestation in the present study, special attentions being paid to CO production in CRC patients by measuring the circulation carboxyhemoglobin (COHb), which is an easy and economic way to reflect the CO in circulation. Accordingly the possibility of COHb as an indicator of CRC was evaluated. In addition, HO-1 expression is validated to be potently increased in response to radiotherapy and chemotherapy in various tumors, which is associated with resistance to anticancer therapy [31,32]. Therefore, the effect of HO-1 on therapeutic sensitivity of conventional anticancer drugs was also tickled in a variety of colon cell lines.

## Methods

### Patients and tissue specimens

Between January 2010 and December 2012, 118 patients, including 63 males and 55 females, received resection for colorectal cancer and polyps at China Medical University Sheng Jing Hospital, were collected in this study. The age of these patients ranged from 37 to 87 (average age, 61.4) years old. None of the patients had received chemotherapy or radiation therapy prior to surgery. The depth of tumor invasion, histological grade and status of lymph node metastasis were obtained from histopathological reports. The staging of CRC was classified using the seventh edition of the International Union Against Cancer Tumor-Node-Metastasis (TNM) staging system. Among these patients, 88 patients underwent radical resection, whereas the others underwent palliative resection because of distant metastasis and positive resection margin. After resection, the specimens were processed routinely for histopathological assessment. Formalin (10%)-fixed, paraffin-embedded specimens from these 118 patients and 65 matched non-tumoral adjacent parenchyma were analyzed. Both the protocol and the use of human tissues were approved by the ethics committee of China Medical University Sheng Jing Hospital, and the patients gave informed consent. The information of patients is summarized in Table 1.

**Table 1:** Summary of patients characteristics involved in the study (n = 118)

**Characteristics no. of patients (%)**	
**Age, years (range)**	37-87
**Sex**	
Male	63
Female	55
**Depth of wall invasion**	
T1	7
T2	17
T3	4
T4	90
**Histological type**	
Well differentiated adenocarcinoma	81
Moderately differentiated adenocarcinoma	28
Poorly differentiated adenocarcinoma	7
Mucin adenocarcinoma	2
**Lymph node metastasis**	
Negative	75
Positive	43
**TNM stages**	
I	18
II	54
III	40
IV	6

### Immunohistochemical staining and evaluation

Tissue specimens were cut into 4-μm thick slices and immunostained by the horseradish peroxidase streptavidin-biotin detection system. Briefly, the samples were deparaffinized in xylene and rehydrated through a graded alcohol series, followed by treatment of 0.3% hydrogen peroxide for 30 min at room temperature to quench endogenous peroxidase activity. Then the samples were incubated in antigen retrieval solution at 37°C for 10 min to improve antigen activity. After incubation with serum blocking solution at 37°C for 30 min, the samples were incubated with primary rabbit anti-HO-1 antibody (Santa Cruz Biotechnology, Inc., Dallas, TX; sc-10789, dilution: 1:160) at 4°C over night, followed by bioinylated secondary antibody for 20 min and then peroxidase-conjugated streptavidin for 20 min at 37°C. The samples were then visualized by DAB solution.

Immunostained sections were evaluated by NIS-Elements Br 3.0 image analysis software. Two pathologists in China Medical University Sheng Jing Hospital who have not informed of patients’ details evaluated the staining of tissue sample. HO-1-positive grade was determined based on the proportion of stained cells on a scale of negative to strong: < 5% of stained cells with the grade of 0 as negative; 5-25% of stained cells with grade 1 as weak expression; 25-50% of stained cells with grade 2 as moderate expression; and > 50% of stained cells with grade 3 as strong expression; grades 1–3 were considered as positive. Meanwhile, the average optical density of 5 fields in each sample was also calculated to indicate the grade of HO-1 expression. Localization of HO-1 in each cell (i.e., nucleus or cytoplasm) was examined, and the numbers of CRC with nuclear HO-1 expression or cytoplasmic HO-1 expression were calculated and related to the clinicopathological features of CRC.

### Chemicals

Hemin and protoporphyrin IX were purchased from Sigma-Aldrich (St. Louis, MO, USA). ZnPP was synthesized in DDS research institute, Sojo University according to the previous literature [33]. Pirarubicin (THP) was a generous gift from Meiji Seika Kaisha, Ltd, Tokyo, Japan. Tricarbonyldichlororuthenium(II) dimer (CORM2) was from Sigma-Aldrich (St. Louis, MO). Other chemicals of reagent grade were from Wako Pure Chemicals (Osaka, Japan) and used without additional purification.

### Cell culture

Human colon cell lines HT29, DLD1 and murine colon cancer cell line C26 were kindly provided by Dr. Matsumura of National Cancer Center Hospital East of Japan, and Dr. Ishima of Kumamoto University School of Pharmacy. HT29 and DLD1 cells were maintained in Dulbecco’s modified Eagle medium (DMEM) supplemented with 10% fetal bovine serum (Invitrogen, Carlsbad, CA, USA) and 1% penicillin/streptomycin, and the C26 was grown in RPMI-1640 medium with 10% fetal bovine serum, at 37°C in the presence of 5% CO_2_.

### RNA extraction and reverse transcription PCR analysis

Total RNA was extracted from cultured cells in 6-well culture plates (50000 cells/well) treated by hemin (1 μM) or THP (0.02 μM for C26 cells, 0.6 μM for HT29 cells and 1 μM for DLD1 cells based one the 50% inhibitory concentrations [IC_50_] of THP to each cell) for 24 h, using TRIzol reagent (Invitrogen) according to the manufacture’s instruction. Reverse transcription (RT-PCR) was carried out using High Performance DNA polymerase KOD FX (TOYOBO), with initial denaturation at 95 for 3 min, then 94°C for 30 sec, annealing at 56°C for 30 sec, and extension at 72°C for 30 sec, for 30 cycles. The specific primers for human HO-1, murine HO-1, β-actin and GADPH were as follows: human HO-1, GATGTTGAGCAGGAACGCGAT (forward) and CAGGCAGAGAATGCTGAGTTC (reverse); murine HO-1, GGCCCTGGAAGAGGAGATAG (forward) and GCTGGATGTGCTTTTGGTG (reverse); mouse β-actin, CACAGCTTCTTTGCAGCTCC (forward) and TCTTCATGGTGCTAGGAGCCA (reverse); human GADPH, CATGTGGGCCATGAGGTCCACCAC (forward) and TGAAGGTCGGAGTCAACGGATTTGGT (reverse). PCR products then underwent electrophoresis on 1% agarose gels. The bands of PCR products were analyzed and semiquantified by imaging densitometry (Molecular Imager FX Pro, BioRad Laboratories, Inc., Hercules, CA), as compared with the reference gene (β-actin or GAPDH) PCR amplification products.

### 3-(4,5 Dimethyl thiazol-2-yl) -2,5-diphenyltetrazolium bromide (MTT) assay

The viability of the cells was assessed using MTT assay. Briefly, cells were seeded in 96-well culture plates (3000 cells/well). After an overnight preincubation, cells were treated by different concentrations of THP for 24 h, in the absence or presence of ZnPP (0.5 μM) or hemin (1 μM). After treatment, MTT was added and the cells were incubated for another 4 h. After removal of the supernatant, DMSO was used to dissolve the crystals by agitation for 10 min. The absorbance at 570 nm of each well was read on a microplate spectrometer. Each experiment was done in triplicate. The toxicity of THP was quantified as the fraction of cells surviving relative to untreated controls, and IC_50_ of THP was thus calculated. Similarly, a cytotoxicity assay using an LDH cytotoxicity assay kit (Thermo Scientific Pierce, Rockford, IL) was carried out in C26 cells to compare with results of cell viability from MTT assay.

To verify the effect of ZnPP in this study is mostly due to its inhibitory activity on HO-1, an HO-1 siRNA was used to compare with the results of ZnPP in C26 cells. The sequences of siRNA were 5’-UGAACACUCUGGAGAUGAC-3’ (sense) and 5’-GUCAUCUCCAGAGUGUCCA-3’ (antisense) (Ambion Inc., Austin, TX). C26 cells were preincubated with 30 nM of siRNA by use of TransMessenger Transfection Reagent according to the manufacturer’s directions (Qiagen GmbH, Hilden, Germany) for 24 h, after which the cells were treated with different concentrations of THP as described above. The effect of siRNA treatment on the HO-1 expression of the cells was examined by RT-PCR for HO-1 mRNA as described above, and the cell viability was examined by above-described MTT assay.

### Quantification of circulation COHb and bilirubin in patients

Blood samples of collected 82 CRC patients and 71 non-tumor patients were analyzed by an ABL800 FLEX blood gas analyzer (Radiometer Medical ApS., Denmark), to obtain the COHb concentrations as described as the percentage of total Hb. In addition, circulation bilirubin, another major product of HO, was also measured by an automatic biochemical analyzer (PUZS-300, Perlove Medical Equipment Co., Ltd, Nanjing, China). All the samples were obtained prior to operation.

### Measurement of circulation CO concentrations in mouse C26 colon cancer model

The association of circulation CO with the growth of colon cancer was further examined in a mouse colon cancer C26 model. Female Balb/c mice, 8 weeks old, were obtained from Kyudo Inc. (Saga, Japan), which were maintained under standard conditions and were fed water and murine chow *ad libitum*. All experiments were carried out according to the Guidelines of the Laboratory Protocol of Animal Handling, Sojo University, and were approved by the Animal Care Committee of Sojo University.

Cultured C26 cells (2 × 10^6^) were implanted subcutaneously in the dorsal skin of Balb/c mice. After scheduled times, tumor volume was calculated by measuring longitudinal cross section (L) and transverse section (W) according to the formula V = (L × W^2^)/2. Mice were then killed and blood samples were drawn from the inferior vena cava. To 0.1 ml of blood in a 10-ml glass test tube, 0.4 ml of phosphate buffered saline [PBS(-)] was added. After purging the tube with nitrogen gas, NO donor NOC7 (10 μl) was added to a final concentration of 1 mM. The glass tube was then closed with a silicon stopper and was incubated at room temperature for 2 h during which CO bound to hemoglobin is replaced by NO released from NOC7 because of the higher affinity of NO to hemoglobin than CO. Afterword, 1 ml of the gas in the test tubes was collected and the CO content was measured by using gas chromatography (TRIlyzer mBA-3000; TAIYO Instruments, Inc., Osaka, Japan) equipped with a semiconductor gas sensor.

### Measurement of HO activity in mouse C26 colon cancer model

Tumor tissues collected from C26 tumor-bearing mice at scheduled times after tumor inoculation, were homogenized by a Polytron homogenizer with ice-cold homogenate buffer [20 mM potassium phosphate buffer (pH 7.4) plus 250 mM sucrose, 2 mM EDTA, 2 mM phenylmethylsulfonyl fluoride, and 10 μg/ml leupeptin]. Homogenates were centrifuged at 10,000 × g for 30 min at 4°C, after which the resultant supernatant was ultracentrifuged at 105,000 × g for 1 h at 4°C. The microsomal fraction was suspended in 0.1 M potassium phosphate buffer (pH 7.4) followed by sonication for 2 s at 4°C. The reaction mixture that was used for measurement of HO activity was composed of microsomal protein (1 mg), cytosolic fraction of rat liver (1 mg of protein) as a source of biliverdin reductase, 33 μM hemin, and 333 μM NADPH in 1 ml of 90 mM potassium phosphate buffer (pH 7.4). The mixture was incubated for 15 min at 37°C, at which point the reaction was terminated by the addition of 33 μl of 0.01 M HCl. The bilirubin formed in the reaction was extracted with 1 ml of chloroform, and the bilirubin concentration was determined spectroscopically by measuring the difference in absorbance between 465 and 530 nm, with a molar extinction coefficient of 40 mM^-^1 cm^-^1.

### Statistical analysis

All data are expressed as means ± SD. Data were analyzed by student’s t-test or one-way ANOVA followed by Bonferroni t-test or Fisher's exact test depending on the sample size and type of experiments. A difference was considered statistically significant when *P* < 0.05.

## Results

### HO-1 protein expression pattern in CRC

**Figure 1:**
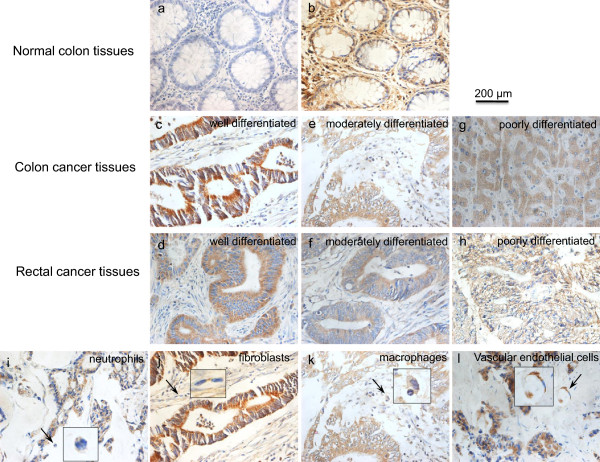
**Immunohistochemical staining of heme oxygenase-1 (HO-1) in colorectal cancer tissues and adjacent normal colon tissues. (a)** and **(b)** HO-1 expressions in normal colon tissues, samples being negative staining and positive staining respectively. **(c)**, **(e)** and **(g)** Examples of HO-1 expressions in colon cancer tissues of different histological differentiation. **(d)**, **(f)** and **(h)** Examples of HO-1 expressions in rectal cancer tissues of different histological differentiation. In colorectal cancers, many stromal cells in including neutrophils **(i)**, fibroblasts **(j)**, macrophages **(k)** and vascular endothelial cells **(l)** are positively stained by HO-1. Arrows and Amplification indicate the representative positive sites.

**Figure 2:**
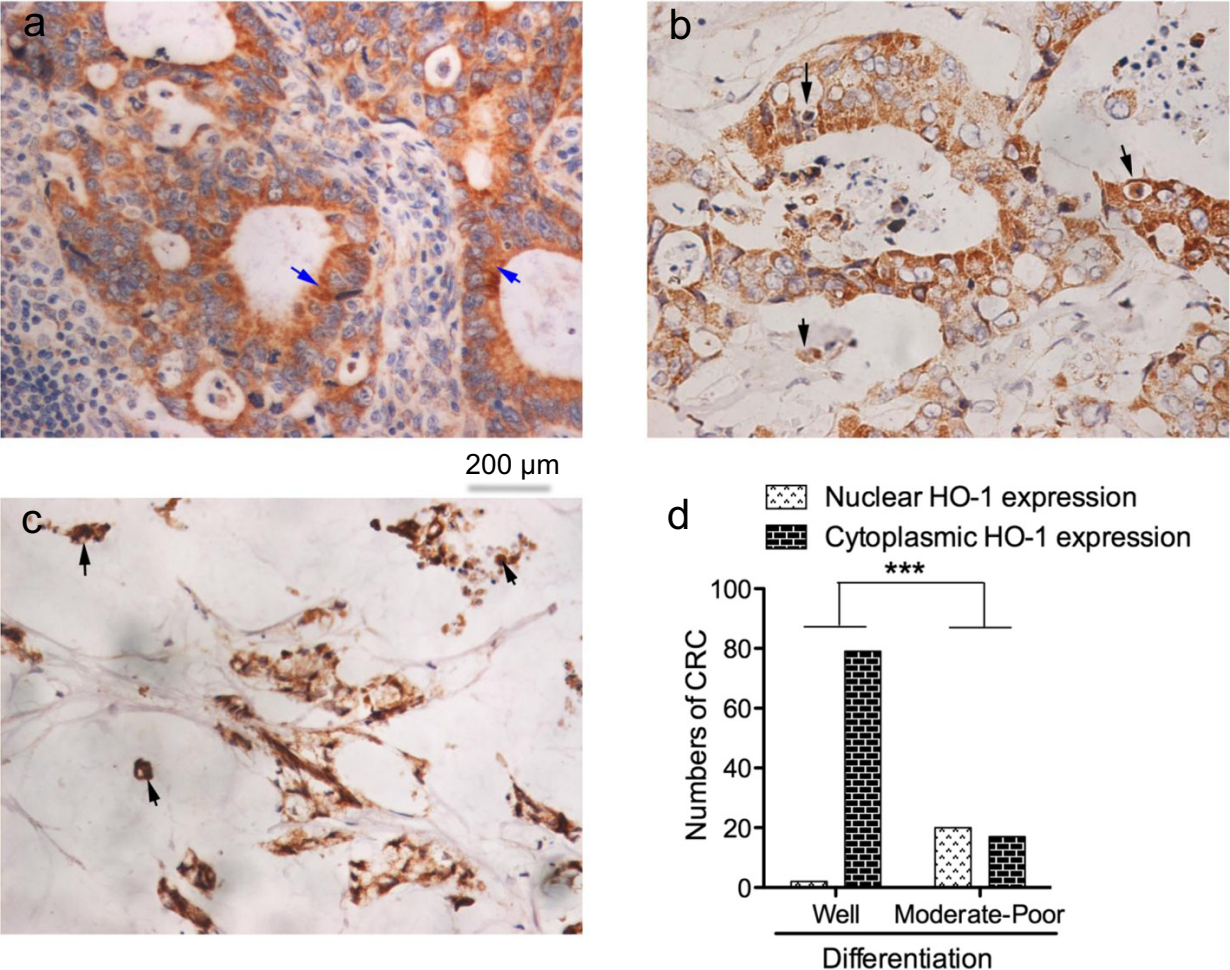
**Localization of heme oxygenase-1(HO-1) in colorectal cancer (CRC) cells. (a)** is an example of well differentiated colon cancer, in which HO-1 expressed mostly in cytoplasm; arrows indicate the cytoplasmic HO-1 staining. **(b)** and **(c)** are examples of moderately differentiated colon cancer and mucinous colon cancer respectively, in which HO-1 was stained largely in cell nucleus; arrows indicate the nuclear HO-1 expression. The numbers of CRC with nuclear or cytoplasmic HO-1 expression are summarized according to different histological differentiation of CRC **(d)**. Data are means ± SD; ***P < 0.001 (Fisher's exact test). See text for details.

Immunohistochemical analysis of surgical resections of CRC tissues and non-tumoral adjacent parenchyma showed that, compared to the relatively lower expressions of HO-1 in adjacent normal colorectal tissues and polyps tissues (11/37, 29.7%; most are grade 0–1) (Figure 1a, b), HO-1 expressed in 72 of 118 tumor tissues (61.0%) with mostly moderate to strong staining (*P* < 0.01; Figure 1c-g, most are grade 2–3). Most polyp tissues, even with atypical hyperplasia, showed the negative staining (Figure 1a, b). In CRC tissues, more obvious HO-1 expression was observed in well differentiated adenocarcinoma (Figure 1c, d, grade 3) than moderately and poorly differentiated adenocarcinoma (Figure 1e-h, grade 1–2). Many stromal cells in tumor tissues including fibroblasts, neutrophils and macrophages were also positively stained for HO-1 (Figure 1i-l).More important, the localization of HO-1 was found significantly different in CRC with different histological differentiation; the majority of well differentiated cancer tissues showed significantly higher cytoplasmic expression of HO-1 than moderately and poorly differentiated tumors which nuclear HO-1 expression was more apparent (Figure 2).

### Correlation between HO-1 expression and clinicopathological features of CRC

**Figure 3:**
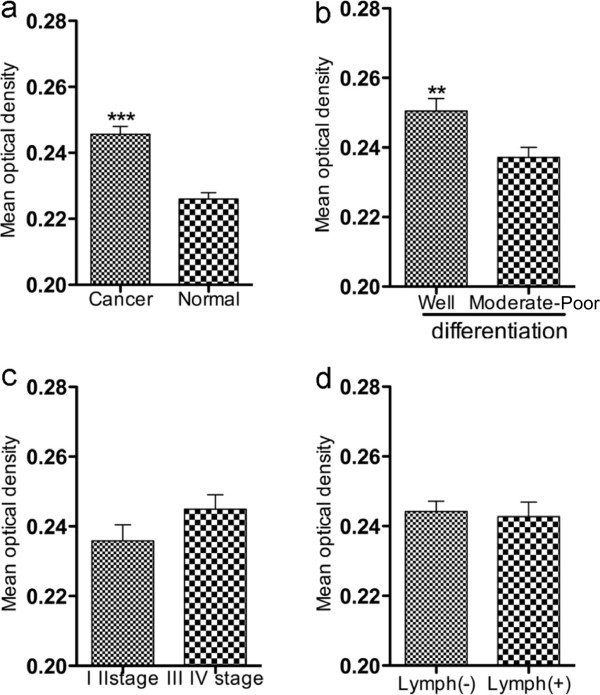
**HO-1 expression intensity in CRC and subcategories of clinicopathological characteristics.** HO-1 expression intensity was evaluated as mean optical density by using NIS-Elements Br 3.0 image analysis system. HO-1 expression was compared between CRC and normal colon tissues **(a)**, between well differentiated tumors and moderately/poorly differentiated tumors **(b)**, between CRC of different TNM stages **(c)** and between tumors with and without lymph node metastasis **(d)**. Values are means ± SD; **P < 0.01, ***P < 0.001 between each compared groups (student’s t-test).

HO-1 protein expression in CRC patients was further evaluated according to the clinicopathological characteristics of CRC, results being summarized in Table 2. Gender is not a determinant factor of HO-1 expression. The HO-1 positive rate was significantly higher in well differentiated cancers (66/85, 81.5%) than moderately/poorly differentiated cancers (22/37, 59.5%; *P* < 0.05). However, no significant correlation was observed in lymph node and liver metastasis, though there is a tendency that CRC patients with HO-1 positive expression trended to accompany with lower rate of lymph node metastasis (Table 2). Neither depth of wall invasion nor TNM stages significantly influence HO-1 protein expression.Consistent with these findings, the mean optical density of HO-1 expression in CRC tissues was significantly higher than that in normal tissue (Figure 3a), similar results were found for well differentiated tumors compared to moderate/low differentiated tumors (Figure 3b); whereas no significant difference was found between tumors of stage I, II and stage III, IV, as well as between tumors with and without lymph node metastasis (Figure 3c, d).

**Table 2:** Heme oxygenamse-1 (HO-1) expression in CRC and its correlation with clinicopathological characteristics

**Characteristics**	**No. of patients**	**HO-1 expression - (%) + (%)**	***P *** **value**
Tissue character			0.0009^***^
CRC	118	46 (39.0) 72 (61.0)	
Non-tumoral adjacent parenchyma	37	26 (70.3) 11 (29.7)	
Sex			0.586
Male	63	26 (41.3) 37 (58.7)	
Female	55	20 (36.4) 35 (63.6)	
Depth of wall invasion			0.441
T1/T2	24	11 (45.8) 13 (54.2)	
T3/T4	94	35 (37.2) 59 (62.8)	
Differentiation			0.011^*^
Well differentiated	81	15 (18.5) 66 (81.5)	
Poorly/moderately differentiated	37	15 (40.5) 22 (59.5)	
Lymph node metastasis			0.079
Negative	75	26 (34.7) 49 (65.3)	
Positive	43	22 (51.2) 21 (48.8)	
Liver metastasis			0.56
Negative	114	45 (39.5) 69 (60.5)	
Positive	4	1 (25) 3 (75)	
TNM stages			0.281
I	18	6 (33.3) 12 (66.7)	
II	54	24 (44.4) 30 (55.6)	
III	40	21 (52.5) 19 (47.5)	
IV	6	1 (16.7) 5 (83.3)	

*P < 0.01; ***P < 0.001, Fisher's exact test.

### Measurement of circulation CO in CRC patients and non-cancer patients

Because the major source of CO (i.e., more than 80%) in mammals is HO-catalyzed heme catabolism [28], and HO-1 is the inducible form of HO responding to various pathological stimuli and stresses, measuring the circulation CO levels may probably a useful way to reflect the expression of HO-1. For this aim, we detected COHb in blood of CRC patients as well as non-cancer patients, because most CO binds to Hb in circulation. Patients with smoking history and chemotherapeutic history were excluded in this study to minimize the external influence.

**Figure 4:**
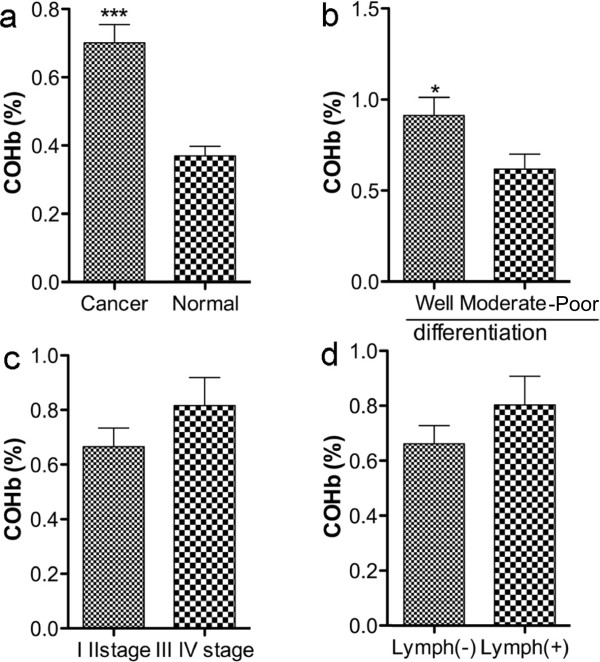
**Circulation CO levels as evaluated as carboxyhemoglobin (COHb) in CRC patients.** Circulation CO levels in CRC patients were analyzed by an ABL800 FLEX blood gas analyzer, and the values were compared between CRC and non-tumor normal patients **(a)**, as well as between different clinicopathological characteristics of CRC as described in Figure 3**(b, c, d)**. Values are means ± SD; *P < 0.05, ***P < 0.001 between each compared groups (student’s t-test). See text for details.

The results were shown in Figure 4. Well-consistent with the findings of HO-1 expression shown in Figure 3 and Table 2, CRC patients showed significantly higher COHb levels than non-cancer patients (*P* < 0.001, Figure 4a). COHb levels in well differentiated CRC patients were markedly higher than those in moderately/poorly differentiated CRC patients (Figure 4b), whereas COHb levels were not relevant to lymph node metastasis and TNM stage (Figure 4c, d).

Because bilirubin is also an important product generated by HO-1, we also measured the circulation bilirubin levels in some CRC patients compared to non-tumor patients. However, no elevated bilirubin levels were observed (Additional file 1: Figure S1), which did not correlate with the results of COHb. This may probably due to that the circulation bilirubin level does not only reflect heme degradation, but is largely influenced by liver function, thus it may be difficult to evaluate in vivo HO-1 activity by circulation bilirubin levels.

### Increasing circulation CO levels in C26 solid tumor-bearing mice accompanied with tumor growth

**Figure 5:**
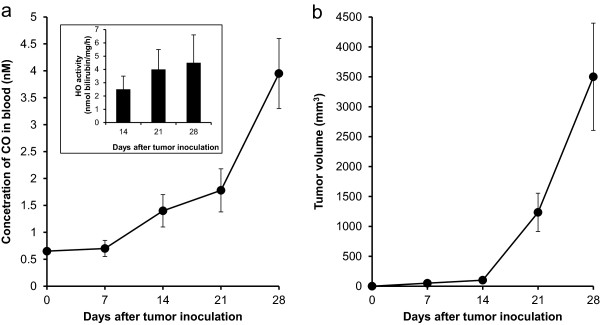
**Increasing blood CO concentrations in C26 solid tumor-bearing mice accompanied with tumor growth and increased HO activity in tumor.** Murine colon cancer C26 solid tumor was obtained by injecting cultured C26 cells (2 × 10^6^) subcutaneously in the dorsal skin of Balb/c mice. After scheduled times, CO concentrations in circulation was detected by gas chromatography **(a)**, and tumor volume was measured **(b)**. Inset of **(a)** shows the HO activity in tumors at different times after tumor inoculation. Data indicate that the blood CO concentrations increase in parallel with tumor growth. See text for details.

To further clarify the association of circulation CO levels with tumor, we then examined the CO concentrations in blood by use of gas chromatography in a mice colon cancer C26 solid tumor model. As showed in Figure 5, CO concentrations in blood of C26 tumor-bearing mice started to increase from 7 days after tumor implantation, increase of CO continuing in parallel with tumor growth that was described by the size of tumors (Figure 5b), and partly in parallel with the increased HO activity in tumor (inset of Figure 5a). Whereas, no increase of CO was observed in blood of tumor free control mice (data not shown). These supported the results found in CRC patients as shown in Figure 4.

### Importance of HO-1 on the chemotherapeutic sensitivity in colon cancer cells

**Figure 6:**
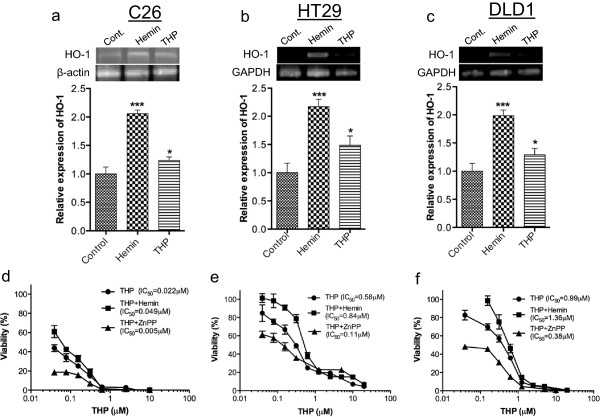
**Induction of heme oxygenase-1 (HO-1) mRNA expression in different colon cancer cell lines by hemin and anticancer drug pirarubicin (THP) (a-c), and the affect of HO-1 on chemosensitivity of these cells to THP (d-f). (a)** C26 cells were treated with 1 μM hemin (HO-1 inducer) or 0.01 μM of THP for 24 h followed by the RT-PCR assay of HO-1; data are presented as relative HO-1 expression compared to the HO-1 expression in control cells without hemin or THP treatment. Similar experiments were performed in HT29 cells **(b)** and DLD cells **(c)**. **(d)** C26 cells were treated with different concentrations of THP in the presence/absence of hemin (1 μM) or HO inhibitor ZnPP (0.5 μM); cell viability after treatment was determined by MTT assay, and the data was described as the percent viability of cells under different treatments. Similar experiments were performed in HT29 cells **(e)** and DLD cells **(f)**. Values are means ± SD; *P < 0.05, **P < 0.01 vs control group (one-way ANOVA followed by Bonferroni t-test). See text for details.

To further evaluate the functional aspects regarding HO-1 expressed in colon cancer cells, we investigated its potential affects on chemotherapy with the use of THP. Over-expression of HO-1 was induced by hemin, and ZnPP was utilized to inhibit HO-1. Either ZnPP or hemin, at the dose used in the present study (i.e., 0.5 μM and 1 μM respectively) did not affect the cell viability (Additional file 1: Figure S2). Hemin (1 μM) significantly induced the HO-1 expression in all tested colon cancer cell lines (Figure 6a-c). In parallel with the increase of HO-1, the cytotoxicity of anticancer drug THP as examined by MTT cell proliferation assay was remarkably inhibited, i.e., the IC_50_ of THP was elevated to 1.4-2.2 folds in different cells (Figure 6d-f). Moreover, when cells were treated by anticancer drug THP, significant increase of HO-1 expression could also be found, though the effect was less potent than hemin (Figure 6a-c), indicating that during chemotherapy cancer cells may upregulate HO-1 to defend against chemotherapeutic attack. Increased vulnerability of C26 cells to THP was similarly seen when CO donor CORM2 was combined with THP (Additional file 1: Figure S3a). All these data suggest the effect of hemin is probably through HO-1 induction and thus CO production. In contrast, when cells were treated by THP in combination with HO inhibitor ZnPP, significantly increased cytotoxicities (i.e., 2.6-5.3 folds compared to cells without ZnPP treatment) were achieved (Figure 6d-f). Similar results were also obtained when LDH cytotoxicity assay was performed (Additional file 1: Figure S4).

In addition, because ZnPP is known to show HO-1 independent effect, its effect on inhibiting HO-1 activity in this study was confirmed by use of HO-1 siRNA. The siRNA used in this study was 30 nM which did not show apparent effect on cell viability, but induced remarkable suppression of HO-1 expression, consequently resulting in significantly increased susceptibility of cells to THP which was similar to those seen in ZnPP treatment (Additional file 1: Figure S3b).

## Discussion

HO-1 and its metabolic product-CO are now known to be involved in many physiological and pathophysiological processes, and related to many diseases and disorders including cardiovascular diseases, inflammation, microbial infection, ischemia-reperfusion injury as well as cancer [28,32,34,35]. The induction and importance of HO-1 in tumors were verified in murine tumor models in 1990s [16], and by now ample studies reported the upregulated expression HO-1 in many cancer cells [11,16-22], and suggested the involvement and importance of HO-1 in the formation, progression, metastasis and prognosis of cancers [36-38]. Association of HO-1 with cancers was also confirmed in many clinical cancers including gastric cancer, pancreatic cancer, breast cancer, prostate cancer, renal cell carcinomas, oral squamous cell carcinomas, nasopharyngeal carcinomas, melanoma and brain tumors [11,20,21,39-44]. However, few studies focused on the roles of HO-1 in CRC especially clinical studies. The present study reported for the first time, the clinical features of HO-1 and CO in CRC patients.

By an immunochemical assay, we found that the HO-1 was expressed in tumor tissues by 72 of 118 (61%) CRC tissues, whereas much lower expression of HO-1 was observed in adjacent non-tumoral tissues and polyp tissues (11 of 37, 29.7%, *P* < 0.01, Table 2). These findings were consistent with the outcome got from a recent clinical study in gastric cancer patients to some extent, which showed a relatively high expression of HO-1 in human gastric cancer tissues (83.8%), compared to those in adjacent non-tumoral gastric tissues (43.8%) [43]. Accordingly, similar to most previous literatures as described above, upregulation of HO-1 seems to be one of typical biological features of CRC.

In view of the clinicopathological characteristics of CRC possibly related to HO-1 expression, the data obtained in this study indicated that the high HO-1 expression in CRC might be associated with the favorable histological differentiation, namely well differentiated tumors exhibited higher rate and intensity of HO-1 expression (Table 2, Figure 3b). Similar results were also found in the recent clinical study of gastric cancers and oral squamous cell carcinomas [21,43]. These results seem to be in contrast to the common notions, namely HO-1 is widely known as a protective molecules in tumor cells against various stress (e.g., oxidative stress from infiltrated leukocytes and macrophages) to support the rapid tumor growth [17,32], thus aggressive and progressing tumors are thought to be accompanied with increased HO-1. However, when we further evaluated the detailed expression pattern of HO-1 in CRC, an interesting and important phenomenon was observed that in contrast to HO-1 expression rate and intensity, much more nuclear expression of HO-1 was found in moderately/poorly differentiated tumors compared to well differentiated tumors (Figure 2). It has been reported recently that nuclear translocation of HO-1 from cytoplasm might be a important scene involved in the protective effects of HO-1 in tumor cells, conferring some mechanisms for tumor growth and progression, for example angiogenesis and drug resistance [45,46]. These results supported, at least partly our findings (Figure 2) that nuclear expression of HO-1 might be essentially correlated with the malignancy of CRC, and strongly suggested that it is critically important to consider the expression pattern of HO-1 in tumor cells when evaluating the roles of HO-1 in tumors. However, further investigations using different types of tumor are needed to clarify the functions and mechanisms of nuclear expression of HO-1 in cancers.

Moreover, no significant associations of HO-1 expression with other clinicopathological features of CRC including invasion, metastasis and TNM stage of tumors, was observed (Table 2). However there was an inclination that negative lymph node metastasis showed higher rate of HO-1 expression, though no significant difference was found (Table 2) and this tendency was not found in the intensity of HO-1 expression (Figure 3d). These findings are partly consistent to other clinical studies using different cancers, for example, Tsuji et al. reported high HO-1 expression in oral squamous cell carcinomas without lymph node metastasis, and no significant correlations between HO-1 expression and tumor sizes as well as staging [21], similar findings were also reported for gastric cancers [43]. HO-1 expression in CRC may thus be helpful to indicate the risk of lymph node metastasis in CRC patients, whereas requiring further studies using large number of samples.

Another important and interesting finding is that, in parallel with the upregulated HO-1 expression in CRC, the circulation levels of CO (measured as COHb) that is majorly derived from HO-1 catalyzed heme metabolism significantly increased in CRC patients (Figure 4), the circulation levels were also consistent with HO-1 expression in different clinicopathological conditions as shown in Table 2 and Figure 3. Because HO-1 does not express only in tumor, it also reflect other pathological conditions such as stress and chemotherapy, in this study patients with history of chemotherapy were excluded to minimize the external influence. As the results of CRC patients were compared with patients with similar characteristics (e.g., age, sex) but only without tumor, we considered the increased COHb was mostly due to the highly expressed HO-1 in tumor, however further investigations using different populations and patients are needed to clarify this point. Furthermore, we confirmed this notion in a murine colon cancer model, showing that circulation levels of CO were increased in parallel with HO-1 activity in tumor as well as tumor growth (Figure 5). These findings strongly suggested the potential of CO being a clinical indicator of CRC, and routinely examining COHb maybe helpful for the diagnosis of CRC and possibly evaluating its prognosis. In addition, when we measured the circulation bilirubin that is another important product generated by HO-1, we did not obtain consistent results with CO, no significant increase of circulation bilirubin levels was observed (Additional file 1: Figure S1). We considered these findings may be due to the complicated metabolism of bilirubin which the excretion of bilirubin is largely affected by liver function, thus CO with relatively simple metabolic pathway may better reflect the functions of HO-1 in vivo.

In addition, as the major product of HO-1, CO is now known as a critical signaling molecule with versatile functions like NO, such as regulation of oxidative stress, modulation of inflammation, cytoprotection/antiapoptosis and vasoactive response [28,32,47,48]. Accordingly therapeutic application of CO either by inhaling CO gas or by use of CO releasing molecules, has been challenged and verified effective in many medical conditions such as inflammation, cardiovascular disease and organ transplantation and preservation [28]. Regarding cancer, most studies indicated CO fulfill mostly the functions of HO-1, e.g., antioxidative and antiapoptotic roles [17,18,32], to defense against the attack from the host such as macrophages and infiltrated leukocytes, and to improve the blood volume of tumor [25], thus supporting the tumor growth. This notion was also supported by the present study, which addition of CO by using CO donor significantly protected C26 cells against the insults of THP that was consistent with the findings using hemin to induce HO-1 (Additional file 1: Figure S2a, Figure 6d). However, a recent study showed that exogenous CO could inhibit the progress of prostate cancer by a mechanism other than HO-1 to induce the metabolic exhaustion of tumor cells [49]. The functions of CO in cancer seems a double-edged sword like ROS such as NO, namely endogenous moderate levels of CO from HO-1 plays mainly a protective/supporting role, whereas exogenously excess CO may trigger the death of cancer cells potentially behaving as a anticancer agent.

In view of the mechanisms involved in the roles of HO-1 in cancers, besides the antioxidative, antiapoptotic effect as described above [17,18,32], many factors involved in the growth and invasion of tumors such as vascular endothelial growth factor (VEGF) and matrix metalloproteinases (MMPs) were reported to be associated with HO-1. For example, VEGF synthesis remarkably decreased after treatment with HO-1 inhibitor in lung carcinoma; inhibition or silencing of HO-1 significantly suppressed MMPs and invasion of lung cancer cells [50,51]. HO-1 is also implicated in the resistance mechanisms of tumors to anticancer therapeutics. Namely, many anticancer drugs and therapeutics, such as anthracycline antibiotics and radiotherapy achieve anticancer effects via induction of ROS and apoptosis, which may induce HO-1 to fight against the cytotoxicity to tumor cells resulting in decreased sensitivity of chemotherapy and radiotherapy. In a clinical study, HO-1 expression was found significantly related to the radiotherapeutic sensitivity of nasopharyngeal carcinomas [42]. Marked induction of HO-1 expression was also found in pancreatic cancer cells upon gemcitabine treatment or radiation; targeted knockdown of HO-1 expression led to growth inhibition of pancreatic cancer cells and made tumor cells significantly more vulnerable to radiotherapy and chemotherapy [31,52]. With regard to CRC, most of advanced CRC are intrinsically resistant to chemotherapy, especially those with metastasis. We thus hypothesized that HO-1 may play a role in drug resistance of CRC, and an in vitro study was carried out in murine colon cell line C26 and human colon cell lines HT29 and DLD1, using anthracycline anticancer drug THP. Similar to the studies described above, induction of HO-1 expression by hemin (Figure 6a-c) significantly elevated the IC_50_ of THP in colon cancer cells (Figure 6d-f), which supported the association of overexpression of HO-1 with drug resistance of cancer cells. More important, the mRNA expression of HO-1 was also increased after THP treatment, suggesting the cytotoxicity of THP may partly attenuated by the upregulated HO-1 (Figure 6a-c). In fact, with the use of HO inhibitor ZnPP, the IC_50_ of THP in each cell line dramatically decreased compared to THP alone group (Figure 6d-f). These findings clearly supported the notions of the involvement of HO-1 in the drug resistance mechanisms of CRC, and suggested the possibility of specific inhibition of HO-1 expression as a new candidate in the cancer therapy of CRC, especially as a sensitizer to chemotherapy and radiotherapy.

## Conclusions

Taken together, the results in the present study indicate HO-1/CO is a useful indicator for CRC. Our findings provide evidence supporting the potential value of COHb examination in clinic as a convenient and easy tool for the diagnosis and prognostic evaluation of CRC, and suggesting that inhibition of HO-1 may be a option to enhance the chemotherapeutic effects of conventional anticancer drugs toward CRC.

## Competing interests

The authors declare that they have no competing interests.

## Authors’ contributions

All authors participated in design of the study. HY and QS performed the clinical studies. HY, JF and LL performed in vitro experiments. HY, JF and HM contributed to data analysis and interpretation. HY, JF and QS conceived of the study, participated in the experimental design, and prepared the manuscript. All authors read and approved the final manuscript.

## Authors’ information

HY and QS are surgeons of Department of General Surgery, Sheng Jing Hospital, China Medical University, and QS is the Director and Professor of the Department, both of them are experts in the diagnosis and surgical treatment of CRC. JF and HM are Associate Professor and Professor in Faculty of Pharmaceutical Sciences/DDS Research Institute Sojo University, who have been working on the association of ROS with cancer for decades, and found the important roles of HO-1 to rapid tumor growth in experimental solid tumors in 1990s. HY has worked in JF and HM’s laboratory as a research fellow in 2013 mainly focusing on the roles of HO-1/CO in cancer and inflammatory diseases and its therapeutic potentials.

## Pre-publication history

The pre-publication history for this paper can be accessed here:

http://www.biomedcentral.com/1471-2407/14/436/prepub

## Supplementary Material

Additional file 1: Figure S1 circulation bilirubin levels in CRC patients and non-tumor patients. Circulation bilirubin levels in CRC patients were analyzed by an ABL800 FLEX blood gas analyzer, and the values were compared between CRC and non-tumor. Values are means ± SE. See text for details. **Figure S2.** Cytotoxicity of ZnPP **(a)** and hemin **(b)** on C26 cells. C26 cells were treated with different concentrations of ZnPP or hemin; percent of dead cells after treatment was determined by MTT assay. Values are means ± SD. **Figure S3.** Effect of carbon monoxide **(a)** and heme oxygenase-1 (HO-1) siRNA **(b)** on chemosensitivity of C26 colon cancer cells to THP. C26 cells were pretreated with HO-1 siRNA (30 nM) for 24 h, or carbon monoxide releasing molecule (CORM2, 10 μM) for 1 h, and treated by THP for 48 h. Cell viability after treatment was determined by MTT assay, and the data was described as the percent viability of cells under different treatments. Vehicle indicates the result for cells treated with TransMessenger Transfection Reagent only (without siRNA); siRNA indicates the result for cells transfected with siRNA for HO-1 mRNA. Inset of (b) shows the results of RT-PCR of HO-1 in C26 cells treated by HO-1 siRNA. Values are means ± SD. *P<0.05, **P<0.01. See text for details. **Figure S4.** Cytotoxicity assay of THP with/without hemin or ZnPP using LDH cytotoxicity assay kit in C26 cells. C26 cells were treated with different concentrations of THP in the presence/absence of hemin (1 μM) or HO inhibitor ZnPP (0.5 μM); percent of dead cells after treatment was determined by LDH cytotoxicity assay. Values are means ± SD. Click here for file
