# Supplementary material for: Upregulation of heme oxygenase-1 in colorectal cancer patients with increased circulation carbon monoxide levels, potentially affects chemotherapeutic sensitivity
Source: BMC Cancer. 2014 Jun 14;14:436. doi: 10.1186/1471-2407-14-436 (PMC4075569; doi:10.1186/1471-2407-14-436)
Supplement: Additional file 1: Figure S1 — circulation bilirubin levels in CRC patients and non-tumor patients. Circulation bilirubin levels in CRC patients were analyzed by an ABL800 FLEX blood gas analyzer, and the values were compared between CRC and non-tumor. Values are means ± SE. See text for details. Figure S2. Cytotoxicity of ZnPP (a) and hemin (b) on C26 cells. C26 cells were treated with different concentrations of ZnPP or hemin; percent of dead cells after treatment was determined by MTT assay. Values are means ± SD. Figure S3. Effect of carbon monoxide (a) and heme oxygenase-1 (HO-1) siRNA (b) on chemosensitivity of C26 colon cancer cells to THP. C26 cells were pretreated with HO-1 siRNA (30 nM) for 24 h, or carbon monoxide releasing molecule (CORM2, 10 μM) for 1 h, and treated by THP for 48 h. Cell viability after treatment was determined by MTT assay, and the data was described as the percent viability of cells under different treatments. Vehicle indicates the result for cells treated with TransMessenger Transfection Reagent only (without siRNA); siRNA indicates the result for cells transfected with siRNA for HO-1 mRNA. Inset of (b) shows the results of RT-PCR of HO-1 in C26 cells treated by HO-1 siRNA. Values are means ± SD. *P<0.05, **P<0.01. See text for details. Figure S4. Cytotoxicity assay of THP with/without hemin or ZnPP using LDH cytotoxicity assay kit in C26 cells. C26 cells were treated with different concentrations of THP in the presence/absence of hemin (1 μM) or HO inhibitor ZnPP (0.5 μM); percent of dead cells after treatment was determined by LDH cytotoxicity assay. Values are means ± SD. [file 1471-2407-14-436-S1.pptx]

## Slide 1
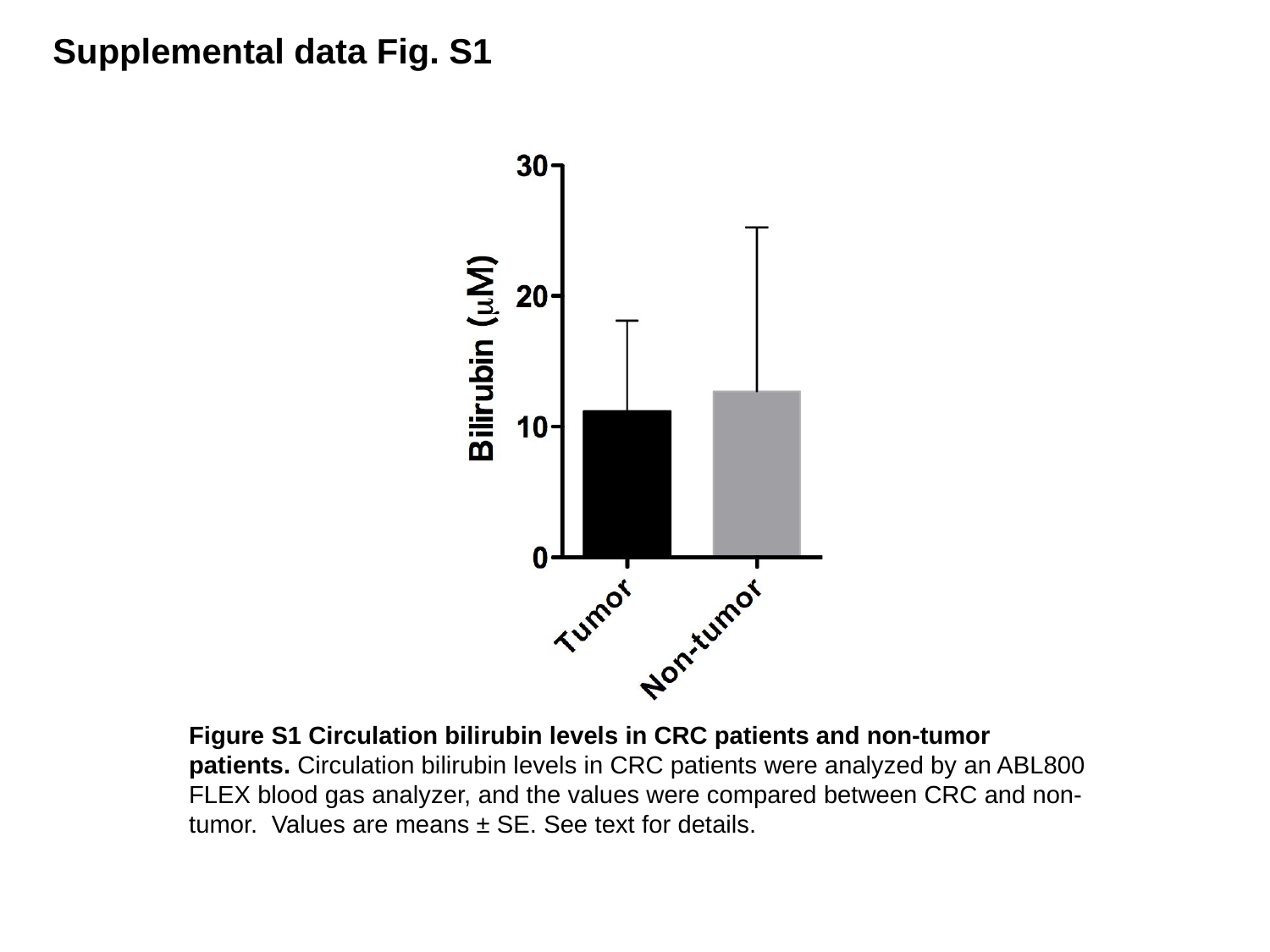

Supplemental data Fig. S1
Figure S1 Circulation bilirubin levels in CRC patients and non-tumor patients. Circulation bilirubin levels in CRC patients were analyzed by an ABL800 FLEX blood gas analyzer, and the values were compared between CRC and non-tumor. Values are means ± SE. See text for details.

## Slide 2
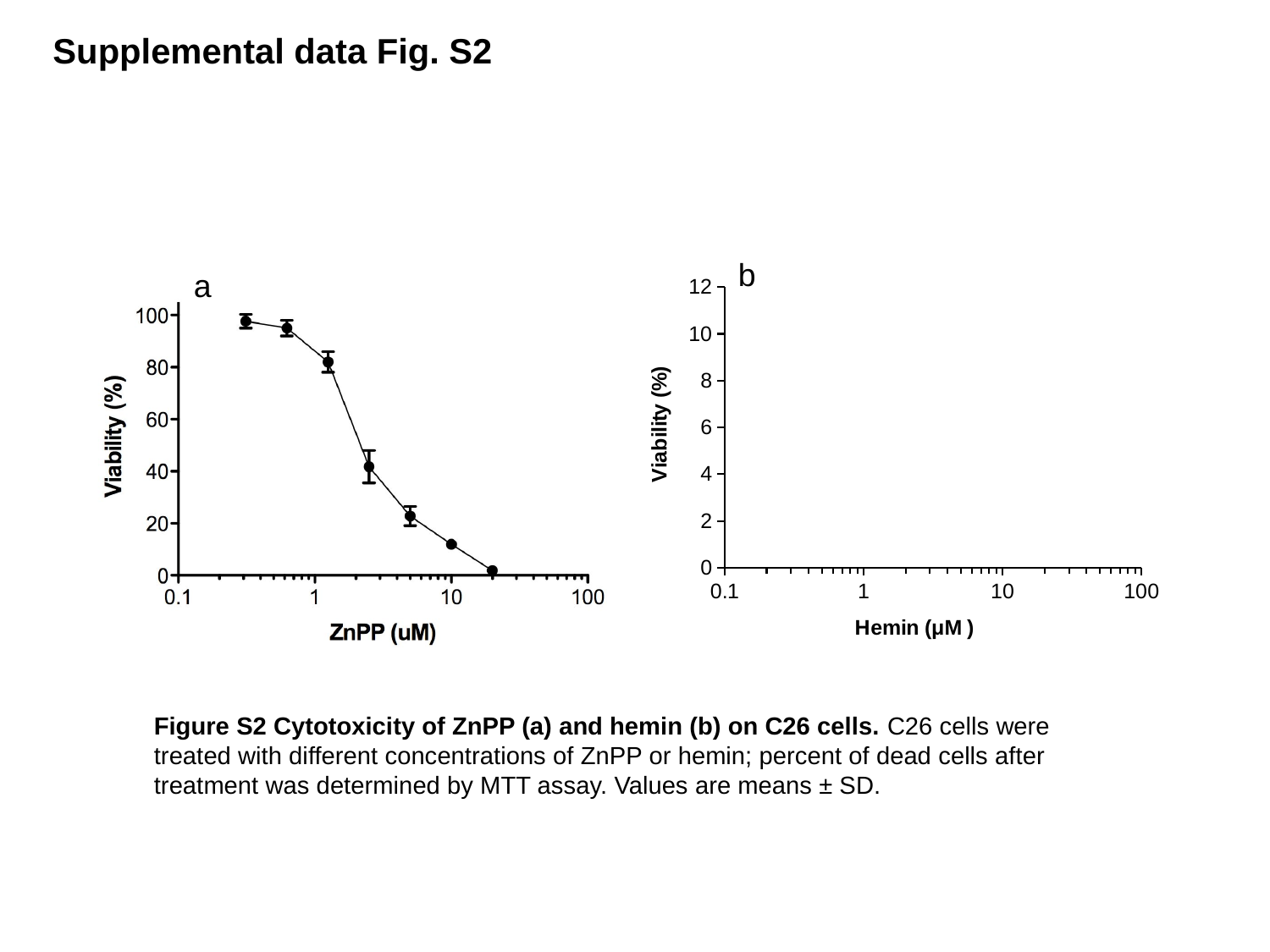

Supplemental data Fig. S2
b
### Chart
| Category | percent survival |
|---|---|a
Figure S2 Cytotoxicity of ZnPP (a) and hemin (b) on C26 cells. C26 cells were treated with different concentrations of ZnPP or hemin; percent of dead cells after treatment was determined by MTT assay. Values are means ± SD.

## Slide 3
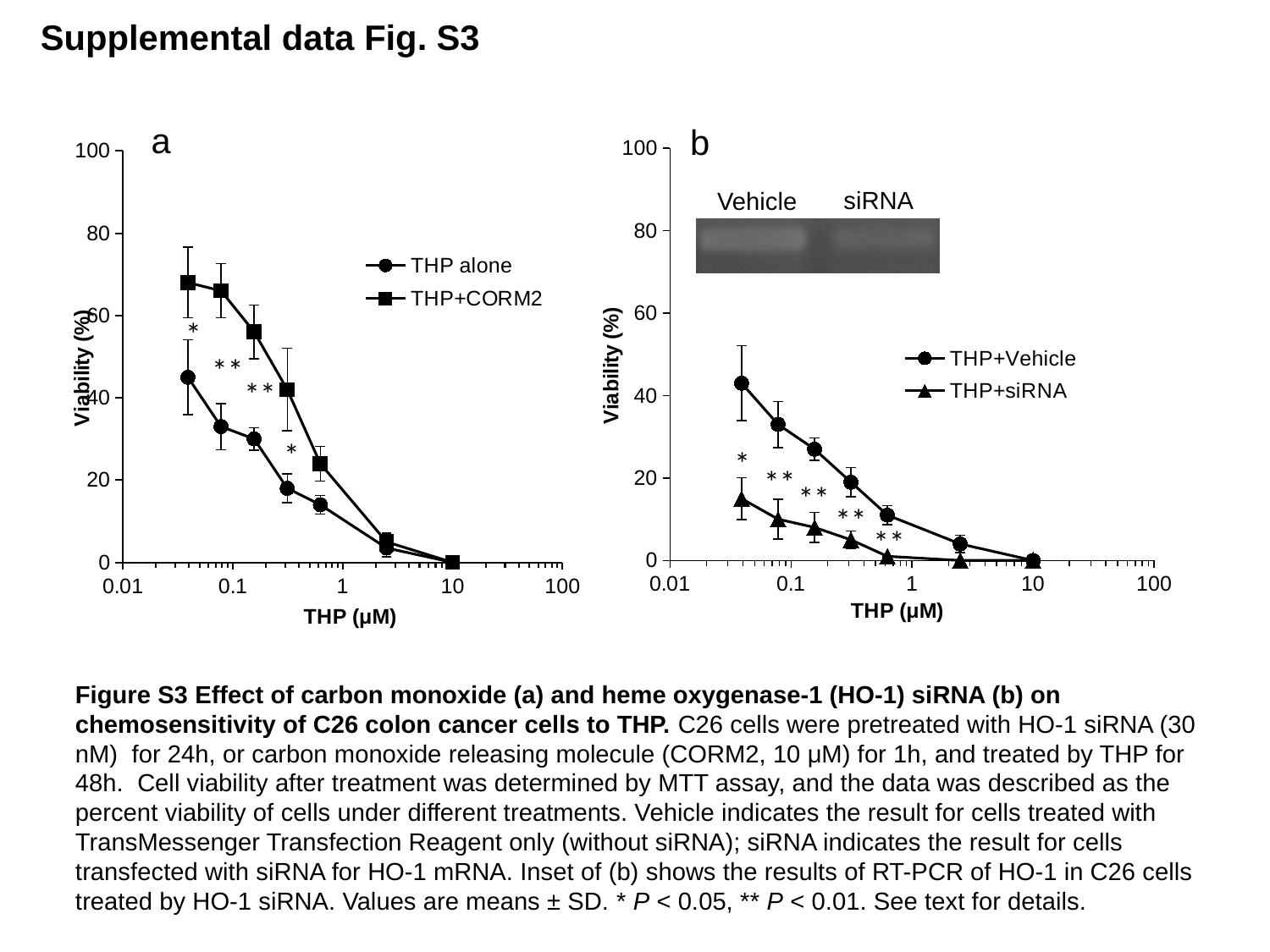

Supplemental data Fig. S3
a
### Chart
| Category | THP alone | THP+CORM2 |
|---|---|---|*
**
**
*
b
### Chart
| Category | THP+Vehicle | THP+siRNA |
|---|---|---|siRNA
Vehicle
*
**
**
**
**
Figure S3 Effect of carbon monoxide (a) and heme oxygenase-1 (HO-1) siRNA (b) on chemosensitivity of C26 colon cancer cells to THP. C26 cells were pretreated with HO-1 siRNA (30 nM) for 24h, or carbon monoxide releasing molecule (CORM2, 10 μM) for 1h, and treated by THP for 48h. Cell viability after treatment was determined by MTT assay, and the data was described as the percent viability of cells under different treatments. Vehicle indicates the result for cells treated with TransMessenger Transfection Reagent only (without siRNA); siRNA indicates the result for cells transfected with siRNA for HO-1 mRNA. Inset of (b) shows the results of RT-PCR of HO-1 in C26 cells treated by HO-1 siRNA. Values are means ± SD. * P < 0.05, ** P < 0.01. See text for details.

## Slide 4
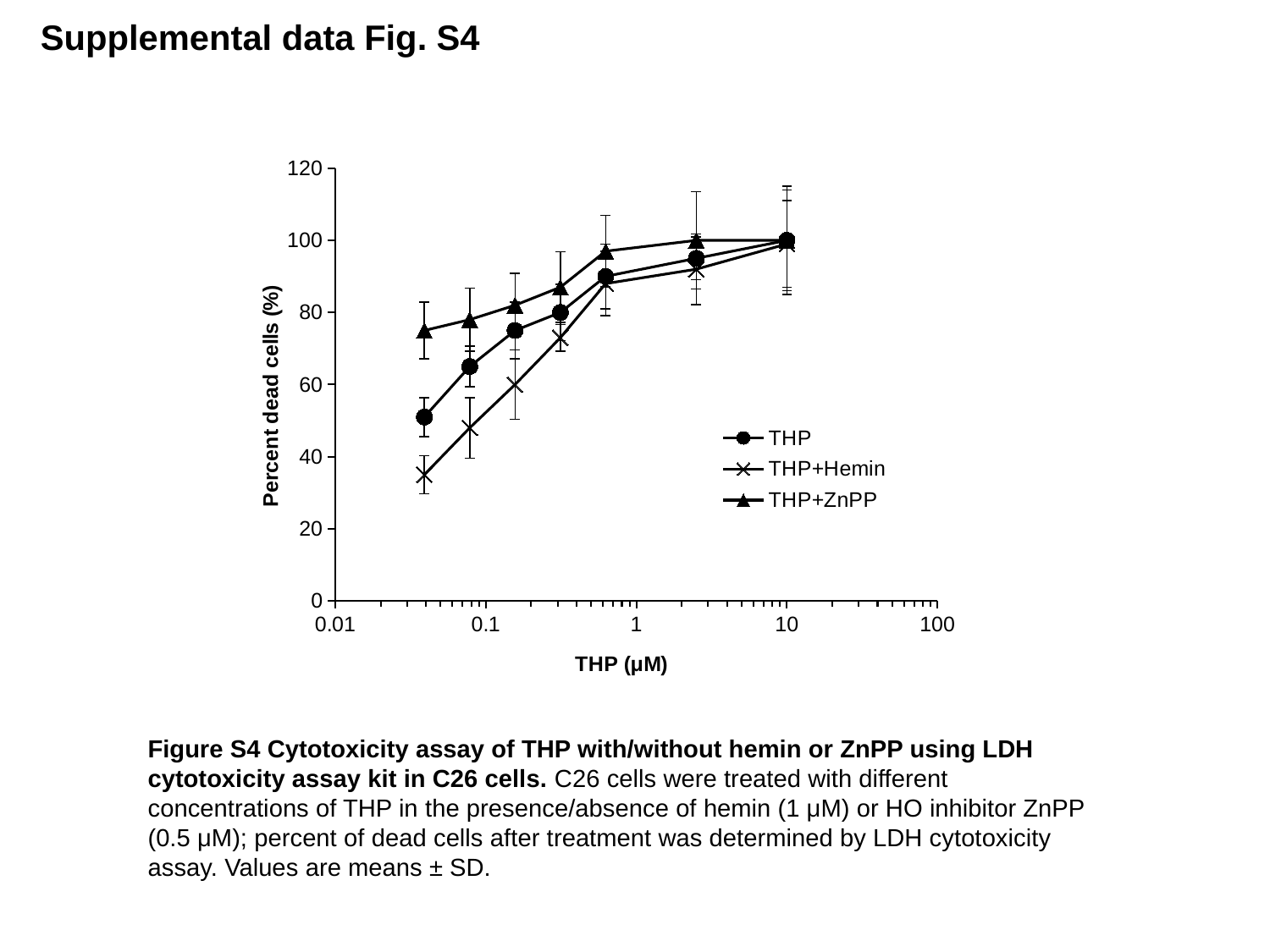

Supplemental data Fig. S4
### Chart
| Category | THP | THP+Hemin | THP+ZnPP |
|---|---|---|---|Figure S4 Cytotoxicity assay of THP with/without hemin or ZnPP using LDH cytotoxicity assay kit in C26 cells. C26 cells were treated with different concentrations of THP in the presence/absence of hemin (1 μM) or HO inhibitor ZnPP (0.5 μM); percent of dead cells after treatment was determined by LDH cytotoxicity assay. Values are means ± SD.
